# Perceived Similarity to Gender Groups Scale: Validation in a Sample of Italian LGB + and Heterosexual Young Adults

**DOI:** 10.1007/s13178-021-00631-5

**Published:** 2021-08-04

**Authors:** Roberto Baiocco, Chiara Antoniucci, Emanuele Basili, Jessica Pistella, Ainzara Favini, Carol Martin, Concetta Pastorelli

**Affiliations:** 1 Department of Developmental and Social Psychology, Faculty of Medicine and Psychology, Sapienza University of Rome, Rome, Italy; 2 Department of Psychology, Faculty of Medicine and Psychology, Sapienza University of Rome, Rome, Italy; 3 School of Social and Family Dynamics, Arizona State University, Tempe, AZ USA

**Keywords:** Gender typicality, Gender similarity, Sexual orientation, Young adulthood, Gender development, Dual identity

## Abstract

**Introduction:**

The present article describes two interrelated studies that examine gender typicality in young adulthood using a new dual-identity approach.

**Methods:**

Participants were recruited online from March 2020 to February 2021 and reported their perceived similarity to own- and other-gender peers as a way to assess their gender typicality. In study 1, the authors conducted an exploratory factor analysis (EFA) and a confirmatory factor analysis (CFA) to test and validate the *Perceived Similarity to Gender Groups Scale* in a sample of Italian young adults (*n* = 571; *M*_age_ = 23.9; SD = 3.60). The authors documented the configural, metric, scalar measurement invariance, and validity. In study 2, the *Perceived Similarity to Gender Groups Scale* adopted in study 1 was used to assess the distribution of different typologies of gender typicality in another sample of Italian young adults who vary in gender and sexual orientation (*n* = 1126; *M*_age_ = 24.3; SD = 3.51).

**Results:**

Results confirmed the structural validity of the *Perceived Similarity to Gender Groups Scale*, revealing the two-factor structure of the scale. Moreover, results of cluster analysis found different typologies of self-perceptions of gender typicality.

**Conclusion:**

Both studies emphasize the relevance of studying gender typicality in young adulthood through a dual-identity approach, highlighting the relevance of gender and sexual orientations.

**Policy Implications:**

The use of the dual-identity approach has significant social and clinical implications as it represents a more flexible and representative model of the complexity of gender typicality.

**Supplementary Information:**

The online version contains supplementary material available at 10.1007/s13178-021-00631-5.

## Introduction

Gender typicality refers to individuals’ perceptions of how typical they believe they are for their gender group across a range of interests, behaviors, activity levels, and the way of interacting with other people (Egan & Perry, 2001; Martin et al., 2017a, b). Recent years have seen significantly increased interest in understanding the relation between gender typicality and young adults’ mental health and behavior (Bukowski et al., 2019). Also, a new, expanded method of assessing gender typicality was recently proposed—a dual-identity approach—in which two types of typicality (called “similarity”) were assessed: feeling similar to one’s own gender and feeling similar to the other gender (Martin et al., 2017a). The dual-identity approach has been explored among children in the United States (Martin et al., 2017a, b) and young adults in the United States (Andrews et al., 2019) and in the Netherlands (Endendijk et al., 2019). Using samples of young adult participants, the goals of the current two studies were the following: study 1: to examine the psychometric properties of the *Perceived Similarity to Gender Groups Scale* (Martin et al., 2017a) by investigating dimensions of own- and other-gender similarity and to explore how these dimensions of gender similarity relate to psychological outcomes; study 2: to investigate the distribution of individuals by gender and sexual orientation in four typologies created from these two dimensions of gender similarity.

Researchers interested in children’s development have described gender identity as a multidimensional construct in which gender typicality is a central component (Braun & Davidson, 2017; Carver et al., 2003; DiDonato et al., 2012; Egan & Perry, 2001; Nielson et al., 2020; Pauletti et al., 2017; Smith & Leaper, 2006; Young & Sweeting, 2004). Despite different studies that examined self-perceptions of gender typicality, the ways it has been theorized and investigated differ. Primary studies considered gender typicality as a single bipolar dimension in which typicality to own gender and typicality to other gender were opposite poles of a continuum (Egan & Perry, 2001).

However, there is a rich theoretical tradition that considered aspects of masculinity and femininity to be two different and independent dimensions: People can identify themselves with one, both, or neither of them, thereby allowing a more nuanced view of what might be considered typical of each gender (Bem, 1981;1985; Costantinopole, 1973; Spence et al., 1975). In line with Bem’s theory, the “dual-identity” approach proposed by Martin and colleagues (2017a, b) represented a new method for measuring gender typicality in which similarity to own gender and similarity to other gender are viewed as independent dimensions, and using these dimensions, individuals can be meaningfully grouped into diverse typologies with different relations to well-being and adjustment. As a result of this conceptualization and measurement of dual identities, four different typologies of children were found to be (a) own-gender similarity (high levels of own-gender similarity and low levels of other-gender similarity); (b) other-gender similarity (high levels of other-gender similarity and low levels of own-gender similarity); (c) both-gender similarity (high levels of own-gender similarity and high levels of other-gender similarity), and (d) low-gender similarity (low levels of own-gender similarity and low levels of other-gender similarity).

Recently, this new approach and measure of gender typicality proposed by Martin and colleagues (2017) was adapted and validated in young adulthood (Andrews et al., 2019; Endendijk et al., 2019). In general, young adulthood is a critical life stage for identity construction and definition and gender identity development (Arnett, 2000; Barret & White, 2002; DiDonato et al., 2012; Leaper & Van, 2008; Lefkowitz & Zeldon, 2006). In these new studies, adults showed less gender similarity to their own gender, and they were more able to identify themselves with other gender than were school-aged children (Andrews et al., 2019; Endendijk et al., 2019). These results are in line with the cognitive-developmental perspective (Erikson, 1968; Kohlberg, 1966), which predicts that adults will show a more flexible perception of one’s own gender thanks to the increased cognitive complexity and perspective-taking as compared to children and adolescents (Barret & White, 2002; Marcell et al., 2011).

## Gender Similarity: Gender and Sexual Orientation Make a Difference

Gender differences in self-perceived gender typicality were well-documented in literature both in childhood (Doescher & Sugawara, 1990; Egan & Perry, 2001; Jewell & Brown, 2014; Menon, 2011; Menon & Hannah-Fisher, 2019; Nielson et al., 2020; Smith & Leaper, 2006; Tam & Brown, 2020; Zosuls et al., 2016) and in young adulthood (Andrews et al., 2019; DiDonato & Berenbaum, 2013; Endendijk et al., 2019; Lefkowitz & Zeldow, 2006) reporting that females showed more flexible attitudes concerning other-gender similarity compared to males (Andrews et al., 2019; DiDonato et al., 2012; Endendijk et al., 2019; Martin et al., 2012, 2017a, b; Zosuls et al., 2016). Furthermore, studies have investigated the relationship between gender typicality, well-being, academic achievement, and psychosocial adjustment, taking into account gender differences (Carver et al., 2003; DiDonato & Berenbaum, 2013; Egan & Perry, 2001; Jewell & Brown, 2014; Lee & Troop-Gordon, 2011; Mehta et al., 2017; Menon & Hannah-Fisher, 2019; Nielson et al., 2020; Ueno & McWilliams, 2010; Yavorsky & Buchmann, 2019). Literature suggests that feeling typical of one’s own gender relates to more gender-typed attitudes, whereas feeling typical of both genders relates to more egalitarian gender attitudes, both in females and males (Andrews et al., 2019; Dinella et al., 2014; Endendijk et al., 2019). Moreover, different studies analyzed the relation between gender-typical behaviors and academic achievement, reporting that adolescents with gender-atypical behaviors have higher academic performance than gender-typical adolescents, especially males (Leaper et al., 2012; Yavorsky & Buchmann, 2019). Regarding sexism, research suggests that males who feel typical of both genders report less sexist beliefs than males who mainly feel typical of their own gender (Andrews et al., 2019; Pauletti et al., 2017). Interestingly, sexist attitudes are found across the spectrum of sexual orientation. The research focused on the relationship between sexism and sexual orientation has suggested that sexual minority people internalize sexist attitudes from a patriarchal society, regardless of their self-interest, attitudes, and behaviors (Cowie et al., 2019; Hässler et al., 2021; Salvati et al., 2018). Additionally, relations with adjustment have been found using gender similarity: Young adults who felt similar to their own gender reported lower social anxiety than other groups (Andrews et al., 2019). In contrast, adults and males who are less typical to their own gender reported the highest level of internalizing problems and externalizing problems, respectively (Endendijk et al., 2019).

Moreover, another line of research has investigated the relationship between gender atypicality and sexual orientation, supporting the hypothesis that lesbian women and gay men are, on average, more gender nonconforming in their interests, appearance, gender-typed behaviors, and feelings, compared to heterosexual women and men, and these patterns hold from childhood through adulthood (Bailey & Zucker, 1995; Dunne et al., 2000; Lippa, 2002, 2008). For instance, Ueno and colleagues (2013) found that gay men, lesbian women, and bisexual people (LGB) tended to have more gender-atypical occupations than heterosexual participants. Green and colleagues (2018) found that LGB adults showed a mixed pattern of gender-atypical self-ratings (e.g., emotional responding, sports interests, interpersonal style, hobbies, and appearance) compared to heterosexual people during their remembered childhoods and in adulthood. A recent study, using photographs in which participants were asked to judge the target’s perceived sexual orientation, happiness, anger, masculinity, or femininity, found that gender typicality facilitates accurate impressions of sexual orientation from both men’s and women’s faces: Less gender-typical individuals were rated as and were found to be more likely gay and lesbian (Bjornsdottir & Rule, 2020).

Research also suggests a complex interaction between gender nonconformity and sexual orientation in predicting psychosocial distress in sexual minority people and in predicting negative attitudes toward LGB people with gender conforming and nonconforming behaviors (Green et al., 2018; Lippa, 2008; Rieger & Savin-Williams, 2012; Salvati et al., 2018). There are two main limitations that constrain understanding of these relationships: (1) studies often used a retrospective approach to investigate the perception of gender typicality in sexual minority people (i.e., recalled childhood gender nonconformity measures, which may be inaccurate); and (2) there has been a limited view of gender typicality in understanding the psychosocial functioning of sexual minority adults. Therefore, the overarching goal of the present research is to assess the usefulness of employing a dual-identity approach to assess gender typicality in young adults varying in gender and sexual orientation. The primary purpose of the first study was to validate a measurement scale that aims to capture gender typicality in a sample of young Italian adults who vary in gender and sexual orientation. To accomplish this goal, we evaluated the psychometric properties of the Italian version of the *Perceived Similarity to Gender Groups Scale* (Martin et al., 2017a) by investigating dimensions of own- and other-gender similarity (Andrews et al., 2019; Endendijk et al., 2019; Martin et al., 2017a, 2017b) and to assess validity by correlating these dimensions with a variety of constructs. Specifically, we included a measure of sexism to assess the validity, and we included several other scales (i.e., self-criticism and life satisfaction) to explore correlations with the two dimensions of similarity. We expect that sexism will relate to own-gender similarity, self-criticism may relate to other-gender similarity, and we will explore relations with life satisfaction.

In the second study, the *Perceived Similarity to Gender Groups Scale* adopted from study 1 was used to identify gender-identity typologies among another sample of young Italian adults. We investigated the prevalence and distribution of the identified typologies in the sample, examining their relations to gender and sexual orientation. Specifically, we hypothesized that (1) male participants, regardless of sexual orientation, will report a higher level of similarity to own gender than female participants; (2) consistent with previous studies about gender atypicality and sexual orientation, we expect that, regardless of gender, heterosexual participants will report a higher level of similarity to own gender than will sexual minority participants (Bjornsdottir & Rule, 2020; Green et al., 2018; Lippa, 2002;2008); (3) we predicted that women, regardless of sexual orientation, will be more represented in both- and other-gender identity typologies compared to men (Andrews et al., 2019); and (4) we expected that lesbian, gay, bisexual, and all other sexual orientations (LGB +) with which persons may identify will report feeling more similar to the other-gender group identity typologies than heterosexual individuals. Finally, considering the paucity of studies investigating gender typicality through a dual-identity approach, we did not hypothesize differences in both- and low-gender typologies depending on sexual orientations.

## Study 1

### Method

#### Procedures

Participants were recruited through online advertisements and an Internet-based survey (hosted by Unipark). Participants were recruited from community recreational centers, universities, and workplaces in Rome, Italy. Since the sexual minority participants were only 10% of the initial sample, other advertisements posted on social networks were directed toward recruiting LGB + people. Participants were not compensated, and participation in the study was voluntary and anonymous. Participants were given 20–25 min to complete the online survey.

Informed consent was obtained from all participants, and those who accepted to take part in the study were given a link to access an Internet-based survey. To meet the inclusion criteria, participants had to (a) self-identified as a sexual minority or heterosexual person, (b) self-identified as a cisgender person, and (c) be of Italian nationality. Based on these criteria, 2 participants were excluded because their nationality was not Italian, and 5 were removed because they self-identified as transgender people. The other 6 participants were not included because they selected “other gender identity,” but they did not specify their identity in the box provided. A total of 99% of distributed questionnaires were completely filled in. Before the data collection begun, the research protocol was approved by the Ethics Commission of the Department of Developmental and Social Psychology of the Sapienza University of Rome (Italy). All procedures performed with human participants were conducted following the ethical standards of the institutional and/or national research committee and with the 1964 Helsinki declaration.

#### Participants

The final sample consisted of 571 (62.9% females; 0.4% intersex) Italian participants with ages ranging from 18 to 32 years (*M*_age_ = 23.9, SD = 3.60). Biological sex was evaluated by the item: “What is your biological sex?” Response options included 1 = female, 2 = male, and 3 = intersex. One participant (0.2%) self-identified as intersex, and we did not include this respondent in the final sample. The sample included individuals who self-identified as heterosexual people (54.8%, *n* = 313; 59.4% women), bisexual people (17.5%, *n* = 100; 96% women), gay men (13.8%, *n* = 79), lesbian women (9.8%, *n* = 56), and other non-heterosexual people (i.e., same-gender-loving, men who have sex with men, women who have sex with women, bi-curious, and questioning; 4%, *n* = 23; 91% women). The ANOVA conducted on age showed that in the present sample, the sexual minority people were younger than the heterosexual people (*F* (1, 569) = 12.590, *p* < .001). The general level of education was medium to high, with 142 participants (24.9%) having at least a university degree and 128 participants (22.4%) having completed secondary school. Concerning socioeconomic status, the majority of individuals, 418 participants (73.2%), reported an average status, whereas 107 (18.7%) reported a below-average status, and 46 (8.1%) declared an above-average status. Demographic distributions are shown in Table 1.

**Table 1:** Sample demographics

	Total sample *N* = 571 (100%)		Heterosexual *N* = 313 (54.8%)		LGB + *N* = 258 (45.2%)	
	*N*	%	*N*	%	*N*	%
Education						
Middle school diploma	29	5.1	9	2.9	20	7.7
High school diploma	272	47.6	131	41.8	141	54.7
Bachelor’s degree	128	22.4	80	25.6	48	18.6
Master’s degree	122	21.4	83	26.5	39	15.1
Postgraduate level	20	3.5	10	3.2	10	3.9
SES						
Extremely low	7	1.2	5	1.6	2	0.8
Low	100	17.5	47	15.0	53	20.5
Average	418	73.2	237	75.7	181	70.2
High	44	7.7	23	7.4	21	8.1
Extremely high	2	0.4	1	0.3	1	0.4
Relationship status						
Single	273	47.8	128	40.9	145	56.2
Engaged (not cohabiting)	210	36.8	134	42.8	76	29.4
Cohabiting	60	10.5	34	10.9	26	10.1
Civil union	2	0.3	0	0	2	0.8
Married	16	2.8	13	4.1	3	1.2
Other	10	1.8	4	1.3	6	2.3

#### Measures

### Perceived Similarity to Gender Groups Scale

Similarity to own-gender and other-gender peers was assessed using Martin and colleagues’ measure (2017a). Participants responded to 10 items indicating how similar they felt to both men and women (e.g., “How similar do you feel to [females/males]?”). Responses ranged from 0 (not similar at all) to 4 (very similar). The original scale presents a two-factor solution in which similarity to female gender (SFG) and similarity to male gender (SMG) are distinct.

### Ambivalent Sexism Inventory (ASI)

Participants completed the 12-item short versions of the Ambivalent Sexism Inventory (Glick & Fiske, 1996; Rollero et al., 2014) and were asked to indicate their agreement or disagreement with each statement on a 0 (strongly disagree) to 5 (strongly agree) scale (sample item, “The world would be a better place if women supported men more and criticized them less”). The items were averaged to a total score, with higher scores indicating a higher level of sexism. Scale descriptives and reliabilities are presented in Table 2.

**Table 2:** Descriptive (means, standard deviations, alphas) of the sample’s scales, divided by gender and sexual orientation

	Total sample *N* = 571 (100%)		Men *N* = 212 (37.1%)		Women *N* = 359 (62.9%)		Heterosexual *N* = 331 (54.8%)		LGB + *N* = 258 (45.2%)	
	*M* (SD)	*α*	*M* (SD)	*α*	*M* (SD)	*α*	*M* (SD)	*α*	*M* (SD)	*α*
Own-gender typicality	2.48 (.83)	.85	2.25 (.85)	.78	2.45 (.82)	.81	2.74 (.78)	.88	2.18 (.78)	.78
Other-gender typicality	1.53 (.78)	.83	1.43 (.83)	.86	1.59 (.75)	.73	1.34 (.76)	.87	1.75 (.74)	.75
ASI	2.28 (.92)	.89	2.59 (.90)	.85	2.10 (.88)	.90	2.64 (.91)	.87	1.85 (.72)	.86
Hated self	2.14 (1.00)	.81	1.93 (.88)	.77	2.27 (1.05)	.82	1.86 (.82)	.76	2.49 (1.09)	.81
Inadequate self	3.39 (1.01)	.87	3.10 (1.01)	.86	3.56 (.98)	.88	3.11 (.98)	.86	3.73 (.95)	.87
SWLS	3.77 (1.35)	.87	3.74 (1.37)	.88	3.79 (1.35)	.86	4.07 (1.29)	.86	3.41 (1.34)	.87

*ASI*, Ambivalent Sexism, *SWLS* Satisfaction with Life Scale

### Self-Criticizing/Attacking Scale

An 8-item version of the Self-Criticizing/Attacking Scale (Gilbert et al., 2004) was used for the present study. To a first probe statement: “When things go wrong for me...” and then either an attacking response to the probe that participants responded to using a 5-point Likert scale ranging from 0 (not at all like me) to 4 (extremely like me). Two subscales were considered in the present study: (1) the Hated Self subscale, which captures a more destructive, disgust-based response to setbacks characterized by self-dislike and an aggressive desires toward the self (sample item, “I have a sense of disgust with myself”) and the (2) Inadequate Self subscale that taps to a sense of feeling of being inadequate facing failures and setbacks (sample item, “I am easily disappointed with myself”). Both subscales showed good reliabilities (see Table 2 for descriptives and reliabilities).

### Satisfaction with Life Scale (SWLS)

The Satisfaction with Life Scale (Diener et al., 1985) comprises 5 items used to measure one’s global satisfaction with life. Each item is rated on a seven-point scale from 1 (strongly disagree) to 7 (strongly agree) (sample item, “If could live my life over, I would change almost nothing”). The results of the five items were summed to produce an overall score that showed good reliability (see Table 2 for descriptives and reliabilities).

#### Data Analytic Plan

To test and validate the structure of the *Perceived Similarity to Gender Groups Scale* in the Italian sample, we conducted exploratory factor analysis (EFA) and confirmatory factor analysis (CFA). The total sample was randomly split into two sub-samples (calibration sample, *n* = 181, for the EFA; validation sample, *n* = 390, for the CFA) using the SPSS random split routine. The data were normally distributed for both calibration and validation samples. One-way ANOVAs were conducted on the two samples to test for significant differences in participants’ demographic characteristics. Results showed no significant differences regarding gender (*F* = 0.22; *p* = 0.88), age (*F* = 2.56; *p* = 0.11), education (*F* = 0.19; *p* = 0.66), and income (*F* = 0.00; *p* = 0.99). Analyses were conducted using Mplus 7 (Muthén & Muthén, 2012). A series of EFAs were conducted on the 10 items to ascertain the goodness of the hypothesized 2-factor solution of the *Perceived Similarity to Gender Groups Scale* (i.e., SFG and SMG) against possible alternative models (i.e., 1- and 3-factor solutions) and select the items with the best psychometric properties. High primary standardized factor loadings were defined as above 0.40, and cross-loadings were defined as having a value ≥ 0.32 (Tabachnick & Fidell, 2013).

Following the EFA, a CFA was conducted on the validation sample to cross-validate the number of factors that emerged from the EFA. Both EFA and CFA models were tested using a maximum likelihood estimator (Kim & Yoon, 2011). Data fit was evaluated through standard fit indices including χ^2^, comparative fit index (CFI), root mean square error of approximation (RMSEA), and standardized root mean squared residual (SRMR) (Hu & Bentler, 1999; McDonald & Ho, 2002). Next, to compare the *Perceived Similarity to Gender Groups Scale* scores across gender and sexual orientation, *configural*, *metric*, and *scalar* measurement invariance (MI) were tested on the factorial structure derived from the CFA. Given the sensitivity of χ^2^ test to sample size, we followed Chen’s (2007) guidelines to consider Δχ^2^ tests and ΔCFI, ΔRMSEA to inspect changes in model fit between nested models. A difference smaller than 0.010 for ΔCFI and 0.015 for ΔRMSEA indicated that the additional constraints were tenable and that MI was supported. Once full or partial scalar MI was established, we tested whether latent means were significantly different between groups. One group was chosen as a reference group with its latent means fixed to zero, whereas factor means of the other group were freely estimated (Schwartz et al*.*, 2014). Finally, bivariate correlations among the study scales were run to test validity with the *Perceived Similarity to Gender Groups Scales*.

### Results

#### Exploratory Factor Analysis

An EFA using Geomin as the oblique method of rotation (Muthén & Muthén, 2012) was conducted to test the dimensionality of the *Similarity scale*. To evaluate if the hypothesized 2-factor structure was appropriate, we ran two preliminary EFAs, in which we extracted 1 and 2 factors. Then, we compared their fit indexes and examined the interpretability of their factor solution to determine the number of factors to be retained. The 2-factor solution χ^2^ (26) = 100.812, *p* < 0.001; CFI = 0.910; RMSEA = 0.126 [90% CI: 0.101, 0.113], SRMR = 0.042 showed a better fit than the 1-factor solution (i.e., Δχ^2^ (9) = 223.07, *p* < 0.001). Although the 2-factor solution provided a better fit to the data, it was still marginally acceptable. As a next step, we deleted those items showing low primary loadings and/or high cross-loadings, and the 2-factor EFA was repeated. In accordance with the original validation study (Martin et al., 2017), items 5 and 6 (i.e., “*look like [girls/boys]*”) were removed due to their high cross-loadings in the two factors. In addition, items 9 and 10 (i.e., “*like to spend time with [girls/boys]*”) presented low loadings and were therefore removed. The remaining 6 items loaded strongly only onto their respective intended factor (see Table 3) and showed an excellent fit χ^2^ (4) = 4.482, *p* < 0.344; CFI = 0.999; RMSEA = 0.026 [90% CI: 0.000, 0.118], SRMR = 0.014. The first factor, labeled *Similarity to female gender* (SFG), taps into individuals’ perception of being similar and typical to women and was defined by three items (factor loadings ranged from 0.61 to 0.91); the second factor, labeled *Similarity to male gender* (SMG)*,* taps into individuals’ perception of being similar and typical to men (factor loadings ranged from 0.69 to 0.81). The factor correlation matrix indicated that SFG and SMG were negatively and significantly correlated at *p* ≤ 0.01.

**Table 3:** EFA factor loadings

		*Similarity to female gender* *(SFG)*	*Similarity to male gender* *(SMG)*
1	How similar do you feel to girls	.80	
3	How much do you act like girls	.81	
7	How much do you like to do the same things as girls?	.69	
2	How similar do you feel to boys		.91
4	How much do you act like boys		.76
8	How much do you like to do the same things as boys?		.62

#### CFA and MI

Next, to the robustness of the identified 2-factor structure, we conducted a CFA on the validation sample. The 2-factor structure fitted the data well (χ^2^ (8) = 12.773, *p* < 0.119; CFI = 0.996; RMSEA = 0.039 [90% CI: 0.000, 0.077], SRMR = 0.020). All items showed high standardized factor loadings (range from 0.70 to 0.90), and both factors showed good reliability coefficients (Table 3). The English and Italian items are presented in Appendix. To compare the scale’s score across the groups at the latent level, we tested the scale’s comparability across gender and sexual orientation by establishing its MI in a multiple-group analytic framework (Millsap, 2012) (see Supplemental File 1 for model fit comparisons).

Regarding MI across gender, the *configural* invariance model fits the data very well (χ^2^ (16) = 27.014, *p* < 0.041; CFI = 0.988; RMSEA = 0.059 [90% CI: 0.012, 0.097], SRMR = 0.039). The further constraints of the factor loadings in the *metric* invariance model showed a worsening in the model fit (i.e., Δχ^2^ (4) = 56.025, *p* < 0.001; ΔCFI =  + 0.058), thereby attesting the need to let some factor loadings vary between males and females. The factor loadings of items 1 and 2 (“*How similar do you feel to [females/males]*”) and 6 (“*How much do you like to do the same things as males*”) were allowed to vary between groups and models’ comparisons gave support to the presence of a partial *metric* invariance (Δχ^2^ (1) = 0.409, *p* = 0.49; ΔCFI = 0.000). Next, *scalar* invariance was tested, and partial invariance was achieved by freeing the intercepts of items 1 (“*How similar do you feel to females*”) and 6 (“*How much do you like to do the same things as males*”) (Δχ^2^ (1) = 0.409, *p* = 0.49; ΔCFI = 0.000).

Then, we compared the scales across gender groups: We found that male adults showed significantly lower latent levels of SFG and significantly higher levels of SMG compared to female adults (*z* =  − 8.81, *p* < 0.001; *z* = 1.47,* p* < 0.001, respectively), showing that males perceived themselves as more typical to the male gender and less typical to female gender compared to females. Regarding sexual orientation, the *configural* invariance model fits the data very well (χ^2^ (16) = 31.626, *p* < 0.011; CFI = 0.987; RMSEA = 0.071 [90% CI: 0.033, 0.107], SRMR = 0.031). Full metric and scalar invariance were also confirmed (Δχ^2^ (4) = 0.409, *p* = 0.98; ΔCFI = 0.003, and Δχ^2^ (4) = 6.188, *p* = 0.18; ΔCFI = 0.004, respectively) supporting the comparability of the *Perceived Similarity to Gender Groups Scale* between heterosexual people and LGB + participants. Latent mean comparisons showed that in the present sample, LGB + individuals showed significantly lower latent levels of SMG than heterosexual individuals (*z* =  − 2.50, *p* < 0.05), showing that LGB + individuals perceived themselves as less typical to the male gender compared to heterosexual individuals. No significant latent mean differences were found on SFG across sexual orientation.

#### Convergent and Divergent Validity

To test for validity, in line with the scale’s conceptualization (i.e., Martin et al., 2017a), scores of the two SMG and SFG factors were re-calculated to reflect the participants’ own- and other-gender similarity (i.e., SMG for men and SFG for women). The two factors were then labeled *own-gender similarity* to reflect the perception of being similar or typical to one’s own-gender and *other-gender similarity* reflecting the perception of being similar or typical to the other gender (see Table 2 for reliability and descriptives). Bivariate correlations were then performed to examine the convergent validity of the *Perceived Similarity to Gender Groups Scale* (i.e., *own-* and *other-gender similarity*) with the Ambivalent Sexism Inventory and the Self-Criticizing/Attacking Scale. Divergent validity was examined through bivariate correlations between the two typicality factors and the Life Satisfaction Scale. Correlations across gender and sexual orientation are presented in Tables 4 and 5.

**Table 4:** Convergent and divergent validity correlations divided for gender

	Males					Females
	(1)	(2)	(3)	(4)	(5)	(6)
Own-Gender Similarity	-	-.13*	.14**	-.13*	-.03	.17**
Other-Gender Similarity	-.56**	-	-14**	.04	02	.01
ASI	.49**	-.39**	-	-.22**	-.21**	.14**
Hated Self	-.24**	.20**	-.03	-	.69**	-.44**
Inadequate Self	-.28**	.26**	-.07	.63**	-	-.43**
SWLS	.23**	-.11	.05	-.36**	.49**	-

*ASI*, Ambivalent Sexism; *SWLS*, Satisfaction with Life Scale

^*^p < .05; ** p < .01

**Table 5:** Convergent and divergent validity correlations divided for sexual orientation

	Heterosexual					LGB +
	(1)	(2)	(3)	(4)	(5)	(6)
Own-Gender Similarity (1)	-	.09*	.05	-.00	.04	.04
Other-Gender Similarity (2)	-.36**	-	-.07	.00	.03	.05
ASI (3)	.30**	-.33**	-	-.15**	-.15**	-.06
Hated Self (4)	-.16**	.08**	-.00	-	.68**	-.36**
Inadequate Self (5)	-.10	.14**	-.04	.61**	-	-.36**
SWLS (6)	.19**	-.00	.04	-.35**	.38**	-

*ASI*, Ambivalent Sexism; *SWLS*, Satisfaction with Life Scale

^*^p < .05; **p < .01

The *own-gender similarity* factor was positively correlated to the ASI score for both males and females and the heterosexual individuals, while no significant association was found for LGB + people. The *own-gender similarity* factor was also negatively associated with *Hated Self* for both male and female adults, while this association was significant only for heterosexual individuals. Furthermore, one negative and significant association was found between the *own-gender similarity* factor and the *Inadequate Self* subscale, but only for males. The *other-gender similarity* factor was negatively correlated to the ASI score for both males and females and the heterosexual individuals, while no significant association was found for the LGB + participants. The *other-gender similarity* factor was also positively associated with Hated Self only for male adults, and positive associations were found between the *other-gender similarity* factor and the *Inadequate Self* only for males and heterosexual individuals.

Regarding divergent validity, the bivariate correlations of the *own-gender similarity* factor were positively correlated to the SWLS score for both males and females and the heterosexual individuals, while no significant association was found for the LGB + participants. To note, regarding the *other-gender similarity* factor, no significant associations were found with SWLS for either male adults or female adults and heterosexual and LGB + individuals.

### Discussion Study 1

The purpose of the present study was to validate a gender-typicality measure in a sample of young Italian adults who varied in gender and sexual orientation. Young adulthood is a critical life stage for defining and further refining one’s identity, and for gender identity, this development is likely related to changing development goals and the social expectancies related to this age (Arnett, 2000; Barret & White, 2002; Marcell et al., 2011). In line with previous studies in childhood and young adulthood (Andrews et al., 2019; Martin et al., 2017a), results of the exploratory factor analysis revealed that the scale is composed of two different factors: (1) similarity to male gender, reflecting the perception of being similar or typical to males, and (2) similarity to female gender, reflecting the perception of being similar or typical to females. Furthermore, the factors are low to moderately negatively related. These results support the dual-identity approach by providing additional empirical support for the structural distinction between one’s own-gender typicality and other-gender typicality (Martin et al., 2017a, b).

The CFA results revealed that the two-factor structure of the *Perceived Similarity to Gender Groups Scale* was the best fit for the data: The comparability of the scale across gender and sexual orientation was supported by *configural*, *metric*, and *scalar* measurement invariance (MI). Correlations with the ASI, *Self-Criticizing/Attacking Scale*, and the SWLS demonstrated convergent and divergent validity. Interestingly, the factorial structure for similarity partially differed from what was found in children (Martin et al., 2017a) and in US adults (Andrews et al., 2019).

Notably, the items which assess similarity in *appearance* (i.e., items 5 and 6 “look like [females/males]”) and that assess social preferences (i.e., “like to spend time with [females/males]”) did not load strongly only into their respective intended factor. While the first result was also found in Andrew and colleagues’ (2019) study, the similarity in hanging out with someone was new. These results could be interpreted in light of the cognitive-developmental perspective, highlighting that increased cognitive maturity and complexity in adults could allow them to think about themselves and their gender typicality more flexibly (Andrews et al., 2019; Endendijk et al., 2019; Erikson, 1968; Kohlberg, 1966). Moreover, gender flexibility both in appearance and hanging out with someone could depict a sign of better adjustment and changing social interests or social expectancies. Adults may have more flexibility in choices of people with whom they spend time as compared to children, given peer group pressures in childhood.

## Study 2

### Method

#### Participants and Procedures

The participants’ recruitment and procedure were the same as in the first study. A total of 1126 (76.1% females) Italian individuals participated in the study. The sample involved heterosexual people (*n* = 319; 64.3% females), heteroflexible people (people who defined themselves as “mostly heterosexual”; *n* = 285; 85.6% females), and LGB + people (*n* = 522; 78.2% females), including bisexual people (*n* = 239), gay men (*n* = 85), lesbian women (*n* = 140), and other sexual orientations (i.e., same-gender-loving, men who have sex with men, women who have sex with women, bi-curious, and questioning; *n* = 58; 86% females). Respondents’ biological sex was investigated through a close-ended question in which they could depict themselves as “male,” “female,” or “intersex.” Two participants (0.2%) defined themselves as intersex: We did not include them in the final sample. Participants’ ages ranging from 18 to 32 years (*M* = 24.3, SD = 3.51). The ANOVA conducted on age showed that in the present sample, heteroflexible people were older than both the heterosexual and LGB + people (*F* (1, 1123) = 17.797, *p* < 0.001). Age, education, SES, and relationship status distributions are reported in Table 6.

**Table 6:** Sample demographics

	Total sample *N* = 1126 (100%)		Heterosexual *N* = 319 (28.3%)		Heteroflexible *N* = 285 (25.3%)		LGB + *N* = 522 (46.4%)	
	*N*	%	*N*	%	*N*	%	*N*	%
Education								
Middle school diploma	54	4.8	8	2.5	9	3.2	37	7.1
High school diploma	517	45.9	141	44.2	103	36.1	273	52.3
Bachelor’s degree	253	22.5	81	25.4	79	27.7	93	17.8
Master’s degree	253	22.4	75	23.5	78	27.4	100	19.2
Postgraduate level	49	4.4	14	4.4	16	5.6	19	3.6
SES								
Extremely low	10	0.9	2	0.6	3	1.1	5	1.0
Low	186	16.5	34	10.7	59	20.7	93	17.8
Average	835	74.2	253	79.3	203	71.2	379	72.6
High	94	8.3	29	9.1	20	7.0	45	8.6
Extremely high	1	0.1	1	0.3	0	0	0	0
Relationship status								
Single	504	44.8	112	35.1	112	39.3	280	53.6
Engaged (not cohabiting)	381	33.8	140	43.9	81	28.4	160	30.7
Cohabiting	176	15.6	51	16.0	63	22.1	62	11.8
Civil union	6	0.6	0	0	2	0.7	4	0.8
Married	34	3.0	14	4.4	16	5.6	4	0.8
Other	25	2.2	2	0.6	11	3.9	12	2.3

#### Measures

### Perceived Similarity to Gender Groups Scale

The *Perceived Similarity to Gender Groups Scale* was adopted from study 1. Scores were averaged to create the two subscales identified in study 1 according to participants’ gender. Both *own-gender similarity* and *other-gender similarity* showed good reliability values (see Supplemental File 2a, b, and c).

#### Data Analytic Plan

To answer our research questions, in line with previous studies (e.g., Andrews et al., 2019; Endendijk et al., 2019; Martin et al., 2017a), a non-hierarchical *k-mean* clustering method was implemented to identify gender-identity typologies among participants. Gender-identity typologies were derived from participants’ responses to the *Perceived Similarity to Gender Groups Scale* (Martin et al., 2017a). Considering that our sample was slightly homogeneous in terms of socioeconomical characteristics, before implementing the clustering analysis, we converted our grouping variables to *z*-scores using the same cluster centers (e.g., Akse et al., 2004; Scholte et al., 2005). We set the number of clusters to four, according to previous works that identified and replicated a four-type solution (e.g., Andrews et al., 2019; Martin et al., 2017a). Thus, to test whether the identified typologies differed on their gender identity, a univariate analysis of variance with subsequent Tukey post hoc test with *p* < 0.001 was performed using the standardized *own-* and *other-gender similarity* scores of our participants (e.g., Akes et al., 2004; Endendijk et al., 2019).

In addition, we investigated the prevalence and distribution of the identified typologies in our sample: We performed contingency table tests, a chi-square test of association, and considered the adjusted standardized residuals, referring to the distribution of gender and sexual orientation in the identified typologies solution. Descriptive statistics for all study variables, including observed means, standard deviations, and correlations, are presented for the total sample (Supplemental File 2a) and differentiated for gender (Supplemental File 2b) and sexual orientation (Supplemental File 2c).

### Results

#### Gender-Identity Typologies

**Fig. 1:**
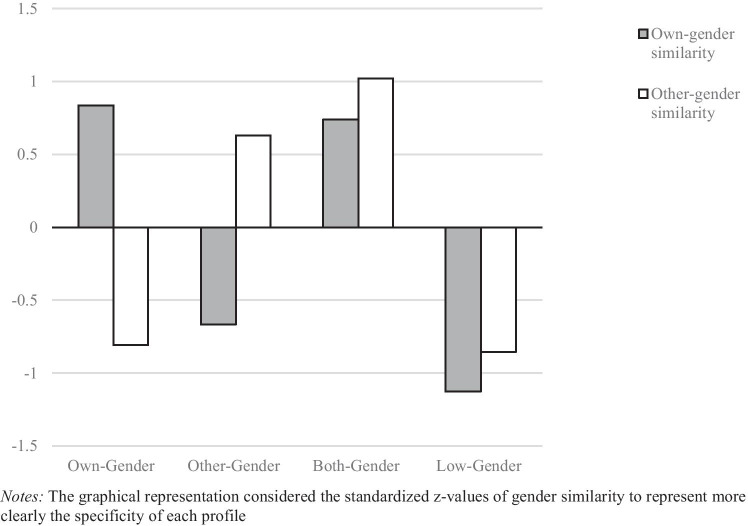
Graphical representation of four different gender-identity typologies

We identified four different types based on participants own- and other-gender similarity, as follows: (1) an *own-gender* profile (*n* = 376; 33% of the sample), characterized by high own-gender similarity, and low other-gender similarity; (2) an *other-gender* profile (*n* = 402; 36% of the sample, the most prevalent profile), characterized by high other-gender similarity, together with low own-gender similarity; (3) a *both-gender* profile (*n* = 185; 16% of the sample), characterized by high levels of both own- and other-gender similarity; and (4) a *low-gender* profile (*n* = 163; 15% of the sample, the less prevalent profile), characterized by lowest levels of both own- and other-gender similarity. Figure 1 shows the graphical representation of the four-typologies solution (*z*-scores).

The univariate analysis of variance attested that each typology significantly differed from other types in their peculiar characteristics. More specifically, as regards the own-gender similarity (*F* (3, 1122) = 740.03; *p* < 0.0001; η^2^ = 0.66; obs. pwr = 1.000), the *low-gender* profile showed the significantly lowest levels of own-gender similarity (mean = 1.43), followed by the *other-gender* profile (mean = 1.80). According to their characteristics, the both-gender and the own-gender profiles showed the highest levels of own-gender similarity (respectively, mean 2.93 and 3.01). Similarly, considering profiles’ levels of other-gender similarity (*F* (3, 1122) = 656.55; *p* < 0.0001; η^2^ = 0.64; obs. pwr = 1.000), the *low-gender* and the *own-gender* profiles showed the lowest levels of other-gender similarity (respectively, mean 0.98 and 1.02). The *other-gender* profile showed medium-to-high levels of other-gender similarity (mean = 2.10), whereas the profile that significantly showed the highest level of this dimension was the *both-gender* profile (mean = 2.39). Overall, these results confirmed the identification of four different gender-identity typologies. Each of these typologies was defined by specific characteristics in terms of how their members perceived themselves as similar or different from people of their own gender.

Regarding the distribution and the prevalence of the four typologies, we found that the most prevalent profile in our sample was the other-gender group (36% of the total sample), followed by the own-gender group (33% of the total sample). The both-gender (16%) and the low-gender (15%) groups represented a small percentage of the total sample. The highest prevalence of the *other-gender* profile could be interpreted in light of the high prevalence of LGB + participants (46% of the total sample).

To deeply investigate these trends, we examined the distribution and the prevalence of our identified typologies using a contingency table, analyzing the chi-square test of association significance and the adjusted standardized residuals. This procedure indicated that considering the gender of our sample, the both-gender and the own-gender groups significantly differed in their distribution (χ^2^ (3, 1122) = 17.108; *p* = 0.001). More specifically, in the both-gender group, there were significantly more females than males (adjRes = 2.7; 18% of females and the 11% of males), and in the own-gender group, there were significantly more males than females (adjRes = 3.7; 43% of males and the 30% of females).

Regarding sexual orientation, all the identified typologies — except the both-gender group — significantly differed in their distribution (χ^2^ (6, 1122) = 86.143; *p* < 0.001). In particular, the own-gender group was significantly more represented by the heterosexual participants than the expected (adjRes = 8.9; 53% of the heterosexual people), and significantly less represented by the LGB + and heteroflexible people than the expected (respectively, adjRes − 5.6 and − 2.8; 25% of heteroflexible participants and 27% of LGB +). Regarding the other-gender group, results attested that there were significantly fewer heterosexual individuals than the expected (adjRes =  − 6.5; the 21% of heterosexual people) and more LGB + participants than the expected (adjRes = 4.6; the 43% of LGB +). At least, in the low-gender group, we found that this profile was significantly less represented by heterosexual participants than the expected (adjRes =  − 2.1; 11% of heterosexual people).

### Discussion Study 2

In line with previous studies investigating gender typicality through a dual-identity approach, the present study aims to recognize four gender-identity typologies based on individual differences in combining own-gender and other-gender similarity. Results from cluster analysis reported similar groups of gender-identity typologies founded in previous studies: own-gender similarity, other-gender similarity, both-gender similarity, and lastly, low-gender similarity (Andrews et al., 2019; Endendijk et al., 2019; Martin et al., 2017a). Interestingly, the percentage of participants in typicality cluster differs from children (Martin et al., 2017a) and other adults’ studies (Andrews et al., 2019; Endendijk et al., 2019). Indeed, we found that the most widespread profile in our sample was the other-gender group (36% of the total sample), followed by the own-gender group (33% of the total sample), both-gender group (16% of the total sample), and low-gender group (15% of the total sample). The higher numbers of young adults who describe themselves as more gender similar to other gender could be explained in several ways. First, this may be due to the increased cognitive maturity that allows adults to be more flexible in their perception of gender roles as compared to children (Andrews et al., 2019; Barret & White, 2002; Kohlberg, 1966; Marcell et al., 2011). Second, the higher prevalence of other gender similar in our sample could be partly explained considering the distribution of the sexual orientation in our sample (46% of the total sample was composed of LGB + people). This result is in line with previous studies highlighting that LGB + people described themselves as more gender atypical than typical (Green et al., 2018).

Another aim of the present study was to describe the distributions of different gender-identity typologies depending on gender and sexual orientation. In line with previous studies, males described themselves as more gender similar to their own gender than females, confirming our first hypothesis. This result could be understood in light of social pressure to conform to gender norms, which is higher for male children/adolescents (Blakemore, 2003; Bukowski et al., 2017; Perry et al., 2019; Ueno & McWilliams, 2010) and for male adults than females counterparts (Andrews et al., 2019; Dinella et al., 2014). Moreover, data showed that females are more represented in the both-gender similar group, while males are more represented in the own-gender similar group. This result partially confirmed our third hypothesis in which we expected that females would describe themselves as more flexible in their gender typicality than males. Females were more highly represented in the both-gender similar group, but we did not find evidence that they feel more similar to the other-gender peer group than males. Given that the both-gender similar typology has been linked to androgyny (Martin et al., 2017b; Pauletti et al., 2017), their strong representation in this group suggests that they hold more flexible attitudes toward own- and other-gender typicality (Martin & Fabes, 2001; Martin et al., 2017a; Perry et al., 2019; Ruble et al., 2007).

Regarding sexual orientation, our results confirmed our fourth hypothesis showing that LGB + individuals were less represented in the own-gender similar profile compared to heterosexual people. Specifically, this result is in line with previous studies highlighting that LGB + people tend to feel more atypical than typical to their own gender (Dunne et al., 2000; Green et al., 2018; Hässler et al., 2021; Lippa, 2002, 2008; Salvati et al., 2018). Generally, results illustrated the value of utilizing this view of gender typicality which considers own- and other-gender similarity as independent and informative dimensions of gender typicality in young adulthood samples.

## Limitation of the Study and Future Directions

Much of the research on gender typicality focuses on children, adolescents, and adults who lived in WEIRD (Western Educated Industrial Rich and Democratic) countries and particularly in the United States and North Europe (Andrews et al., 2019; Egan & Perry, 2001; Endendijk et al., 2019; Martin et al., 2017a). We recognize that what is typical for males and females differs across cultures and intergroup contexts, so cross-cultural research is needed to better understand the definition and development of gender typicality and the different implications of its attribution across cultures. It would be a worthy goal of future research to verify the scale’s validity and explore its usefulness among varying age groups in non-western countries.

The study has some limitations. First, we used a convenience sample, and future studies using other sampling methods and other samples are necessary. Second, the effect of social desirability must be controlled when data are collected with self-report questionnaires. Third, we did not consider the relevance of the variables associated with gender typicality, such as age, socioeconomic status, educational level, ethnicity, and religiosity. Fourth, we did not investigate the effect of the coronavirus disease (COVID-19) pandemic on the self-perception of gender typicality: Future research could deeply investigate this effect using retrospective approaches. Finally, another limitation concerns the composition of participants’ gender identities: Future research may investigate the development and definition of gender typicality, including transgender people and non-binary people, to understand better the complexity of gender typicality and be more representative of the different nuances of gender identity. Ideally, the *Perceived Similarity to Gender Groups Scale* would also be used in longitudinal studies, which allows a closer examination of the trajectories of gender typicality during different life stages, like adolescence and late adulthood, to improve understanding of changes in typicality over different periods of life.

In sum, the present study highlights the relevance of studying gender typicality in young adulthood and of doing so using a broad sample of participants who vary in gender and sexual orientation. Consistent with the early views about gender involving multiple dimensions and akin to today’s views of the importance of understanding intersecting identities, the dual-identity approach better represents the complexity of gender identity and considers the experiences of those who feel similar to both genders or neither (Bem, 1981; Shields, 2008). This conceptualization has important clinical and social implications: It underlines the relevance of approaching gender typicality and gender identity in a more flexible way, moving beyond the gender binary (Martin et al., 2017b). In line with this, it is evident that many adults perceived themselves to be more than simply fitting into male and female categories and instead described themselves as having a more complex, singular, and flexible experience of gender identity (Andrews et al., 2019).

## Policy Implications

The use of the dual-identity approach has significant social and clinical implications as it represents a more flexible and representative model of the complexity of this construct. Being open to individuals expressing a more flexible perception of gender typicality allows one to recognize and understand those who do not identify themselves within a binary vision of gender and helps health and social care professionals increase the quality of care for vulnerable and minority groups (Baiocco et al., 2021). Moreover, this flexible perspective about gender could also have important implications in the school context and in the educational system, promoting more inclusive and safe spaces for all gender identities. Indeed, approaching gender typicality in a plural way at school could allow to recognize different gender identities and expressions, build a more inclusive society, and support youth’s well-being. Thus, more significant efforts are needed to consider gender typicality and its relationship with well-being and social competence, particularly for LGBT + people.

A dual-identity approach to gender typicality helps to redefine a stereotyped vision of gender, opening up toward a plural and inclusive society. We believe that too little attention has been focused on gender typicality during adulthood: This is a relevant topic that was often ignored, despite its significant potential to promote well-being and social competence. We hope this article will help the scientific community promote studies evaluating gender typicality across sexual orientation and gender identity dimensions, especially in countries like Italy, characterized by high levels of social-sexual stigma and negative beliefs regarding the gender people who feel or express themselves as being less typical of their own gender.

### Supplementary Information

Below is the link to the electronic supplementary material.

Supplementary file1 (DOCX 27 KB)

## Data Availability

The data are available upon request to the authors.

## Appendix Revised version of the Perceived Similarity to Gender Groups Scale: English and Italian version

**Table:**

	English version		Italian version
(1)	How similar do you feel to girls?	(1)	Quanto ti senti simile alle donne?
(2)	How similar do you feel to boys?	(2)	Quanto ti senti simile agli uomini?
(3)	How much do you act like girls?	(3)	Quanto ti comporti come le donne?
(4)	How much do you act like boys?	(4)	Quanto ti comporti come gli uomini?
(5)	How much do you like to do the same things as girls?	(5)	Quanto ti piace fare le stesse cosec he generalmente fanno le donne?
(6)	How much do you like to do the same things as boys?	(6)	Quanto ti piace fare le stesse cose che generalmente fanno gli uomini?

Responses ranged from 0 (not similar at all) to 4 (very similar). Subscale scores are computed by averaging subscale item ratings: similarity to female gender (1, 3, 5) and similarity to male gender (2, 4, 6).
