# Supplementary material for: Perceived Similarity to Gender Groups Scale: Validation in a Sample of Italian LGB + and Heterosexual Young Adults
Source: Sex Res Social Policy. 2021 Aug 4;19(3):1270–84. doi: 10.1007/s13178-021-00631-5 (PMC8335452; doi:10.1007/s13178-021-00631-5)
Supplement: Supplementary file 1 — Supplementary file1 (DOCX 27 KB) [file 13178_2021_631_MOESM1_ESM.docx]

**Supplemental File 1.** *MI across Gender and Sexual Orientation*

|  | |  | Measurement Invariance across Gender | | | |  |  |  |  |
| --- | --- | --- | --- | --- | --- | --- | --- | --- | --- | --- |
|  | χ 2 | *df* | CFI | RMSEA | SRMR | Model Comparison | χ2 diff | ∆*df* | ∆*CFI* | ∆*RMSEA* |


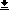
**Model 1**.

| Configural  Invariance | 27.014* | 16 | .988 | .059(.012,.097) | .039 |  |  |  |  |  |
| --- | --- | --- | --- | --- | --- | --- | --- | --- | --- | --- |
| **Model 2**. Metric  Invariance | 83.039** | 20 | .930 | .127 (.100, .156) | .184 | 2 vs 1 | 56.025* | 4 | .058 | -.068 |
| **Model 3**. Partial  Metric Invariance^a^ | 27.489** | 17 | .988 | .056(.000,.093) | .040 | 3 vs 1 | .475 | 1 | .000 | -.003 |
| **Model 4**. Scalar  Invariance | 57.510** | 21 | .960 | .094 (.066,.124) | .077 | 4 vs 3 | 30.021*  * | 4 | .028 | -.038 |
| **Model 4**. Partial  Scalar Invariance ^b^ | 30.433* | 19 | .987 | .056 (.007,.091) | .044 | 4 vs 3 | 2.944 | 2 | .001 | .000 |
|  |  |  | Measurement Invariance across Sexual Orientation | | | |  |  |  |  |
|  | χ 2 | *df* | Model  CFI RMSEA SRMR  Comparison | | | | χ2 diff | ∆*df* | ∆*CFI* | ∆*RMSEA* |
| **Model 1**.  Configural  Invariance | 31.626* * | 16 | .071  .987 .031  (.033,.107) | | | |  |  |  |  |
| **Model 2**. Metric  Invariance | 32.035* | 20 | .056  .990 0.34 2 vs 1  (.010,.090) | | | | 0.409 | 4 | .003 | -.015 |
| **Model 3**. Scalar  Invariance | 38.223* * | 24 | .056  .988 0.36 3 vs 2  (.016,.087) | | | | 6.188 | 4 | -.002 | .000 |

*Notes*: χ ^2^= Chi-square Goodness of Fit; *df* = degrees of freedom; CFI = Comparative Fit Index; RMSEA = Root Mean Square Error of Approximation. All the Δ index comparisons are made with respect to the previous model. * p <= .05; ** p <= .01

^a^ Free factor loadings of items 1 and 2 (“*How similar do you feel to [females/males]*”) and 8 (“*How much do you like to do the same things as boys*”) ^b^ Free intercept of item 8 (“*How much do you like to do the same things as males*”).

**Supplemental File 2a.** *Correlations, descriptive statistics, and reliability of the study 2 variables for the overall sample*

|  | 1 | 2 | 3 | 4 |
| --- | --- | --- | --- | --- |
| 1 Gender (1) | *–* |  |  |  |
| 2 Sexual Orientation (2) | -.12*** | *–* |  |  |
| 3 Own-gender Similarity (3) | .09** | -.26*** | *–* |  |
| 4 Other-gender Similarity (4) | -.11*** | .22*** | -.16*** | *–* |
| Mean | n.p. | n.p. | 2.34 | 1.62 |
| SD | n.p. | n.p. | .80 | .75 |
| Cronbach’s Alpha | n.p. | n.p. | .82 | .78 |

*Note*. * *p* < .05; ** *p* < .01; *** *p* < .001.

**Supplemental File 2b.** *Correlations, descriptive statistics, and reliability of the study 2 variables controlling for gender*

|  |  | 1 | 2 | 3 |
| --- | --- | --- | --- | --- |
|  | Sexual Orientation (1) | *–* |  |  |
|  | Own-gender Similarity (2) | -.25*** | *–* |  |
|  | Other-gender Similarity (3) | .21*** | -.15*** | *–* |
| Female | Mean | n.p. | 2.30 | 1.67 |
|  | SD | n.p. | .80 | .71 |
|  | Cronbach’s Alpha |  | .78 | .67 |
| Male | Mean | n.p. | 2.46 | 1.48 |
|  | SD | n.p. | .78 | .83 |
|  | Cronbach’s Alpha |  | .79 | .80 |

**Supplemental File 2c.** *Correlations, descriptive statistics, and reliability of the study 2 variables controlling for Sexual Orientation.*

|  |  | 1 | 2 | 3 |
| --- | --- | --- | --- | --- |
|  | Sexual Orientation (1) | *–* |  |  |
|  | Own-gender Similarity (2) | .05** | *–* |  |
|  | Other-gender Similarity (3) | -.08** | -.11*** | *–* |
| Heterosexual | Mean | n.p. | 2.69 | 1.33 |
|  | SD | n.p. | .77 | .75 |
|  | Cronbach’s Alpha |  | .90 | .86 |
| Heteroflexible | Mean | n.p. | 2.27 | 1.74 |
|  | SD | n.p. | .77 | .64 |
|  | Cronbach’s Alpha |  | .79 | .68 |
| LGB+ | Mean | n.p. | 2.16 | 1.75 |
|  | SD | n.p. | .77 | .74 |
|  | Cronbach’s Alpha |  | .75 | .66 |
